# A Comparative Assessment of the Efficacy and Toxicity Profiles of Dual and Triple Oral Metronomic Chemotherapy in Advanced Head and Neck Cancers in a Tertiary Care Center

**DOI:** 10.7759/cureus.95490

**Published:** 2025-10-27

**Authors:** Rishi P Nair, Sanjay Santhyavu, Atul Kumar Gupta, Puneet Pareek, Amith Mohan, Depanshu Aggarwal, Antonio Fernandes, Rakesh Vyas, Bharti Devnani, Akanksha Solanki

**Affiliations:** 1 Radiation Oncology, All India Institute of Medical Sciences, Jodhpur, Jodhpur, IND

**Keywords:** head and neck cancer, oral methotrexate, oral metronomic chemotherapy, overall survival, palliative radiotherapy

## Abstract

Background

The standard of care for unresectable or metastatic advanced head and neck squamous cell carcinoma (HNCSCC) is either immunotherapy, cetuximab-based monotherapy, or combination therapy. However, this is often unavailable to most patients due to financial constraints. Existing studies have already demonstrated that oral metronomic therapy (OMT) outperforms other standard chemotherapy options. In this study, we aim to compare the efficacy of dual versus triple OMT in improving overall survival (OS) and progression-free survival (PFS) and assess the factors that affect these outcomes.

Methodology

This retrospective analysis, conducted after receiving approval from the All India Institute of Medical Sciences Institutional Ethics Committee, included patients diagnosed with HNCSCC who received either dual or triple metronomic chemotherapy between 2019 and 2024. The patients received methotrexate 40 mg/m² PO weekly, celecoxib 200 mg twice daily, with or without erlotinib 150 mg daily. Palliative radiotherapy was administered at the physician’s discretion (30 Gy/10 fractions). The primary endpoints were OS and PFS. Secondary endpoints included the assessment of safety and other factors affecting survival. OS and PFS were analyzed using Kaplan-Meier and log-rank tests, with hazard ratios (HRs) estimated by Cox proportional hazard models.

Results

The study included 97 patients, with 45 receiving dual OMT and 52 receiving triple OMT. With a median follow-up of 10.5 months, the median OS was five months in the dual OMT group and seven months in the triple OMT group (HR = 0.56; 95% confidence interval (CI) = 0.33-0.93; p = 0.025). Median PFS was two months in the dual OMT group and 5.6 months in the triple OMT group (HR = 0.29; 95% CI = 0.18-0.47; p < 0.000001). In addition to the regimen (dual vs. triple OMT), age, sex, tumor site, receipt of prior chemotherapy, presence of metastasis, receipt of palliative radiotherapy, whether the disease was de novo or residual/recurrent, and other factors were analyzed for their effect on OS and PFS. The addition of palliative radiotherapy significantly increased both PFS (one vs. four months, HR = 0.4; 95% CI = 0.24-0.67; p = 0.00015) and OS (4.2 months vs. 7.8 months, HR = 0.42; 95% CI = 0.25-0.72; p value = 0.01). The sequence of radiotherapy did not affect OS or PFS. Grade 3 oral mucositis occurred in 5.7% of the triple OMT group and 4.4% of the dual OMT group patients. Only one patient had a Grade 3 rash with erlotinib.

Conclusions

The addition of erlotinib to the dual metronomic chemotherapy regimen demonstrated a significantly improved OS and PFS and a well-tolerated toxicity profile. Palliative radiotherapy quadrupled the PFS and nearly doubled the OS in HNCSCC, even in patients with prior exposure to radiotherapy. These findings suggest a potential benefit in utilizing triple therapy in combination with palliative radiotherapy for specific subgroups of patients with HNCSCC, warranting further prospective clinical trials to validate these results.

## Introduction

Oral cavity cancer, along with lip cancer, is among the most frequently diagnosed cancers in India, comprising 10.2% of all cancer cases in the country, according to GLOBOCAN 2022 data [1]. A large majority of the head and neck cancers in developing countries like India are diagnosed at an advanced stage and are mostly inoperable [2]. Treatment options for locally advanced, recurrent, and metastatic head and neck cancer are primarily palliative. The treatment options for inoperable head and neck cancers include palliative radiotherapy, platinum-based chemotherapy, and immunotherapy [3]. Despite exercising all available treatment options, the prognosis of such advanced-stage cancers is dismal. Further, platinum-based chemotherapy harbors an added toxicity risk and increased hospital visits and admissions. The available immunotherapy options, according to the latest literature, are cetuximab, nivolumab, and pembrolizumab [4-6]. These therapies are also not free of toxicities. Immunotherapy, on the other hand, is expensive and not affordable to the majority of patients in developing countries. The response rate to immunotherapy agents, such as cetuximab, as reported in a 2008 study by Vermoken et al., was only 36%, and the median progression-free survival (PFS) was 5.6 months [4]. Additionally, programmed cell death protein 1 (PD1) inhibitors nivolumab and pembrolizumab, which are currently used as first-line treatment in such tumors with PD1 and programmed death-ligand 1, have shown response rates of only 13-18%. The KEYNOTE-048 study showed that the PFS rate at 12 months was only 17% in the overall patient population treated with pembrolizumab alone or in combination with chemotherapy [5]. These studies suggest that most patients do not benefit from costly treatments, such as immunotherapy, thereby adding to the financial burden. Even with the availability of immunotherapy drugs, the overall survival (OS) is not significantly prolonged.

Frequent administration of low-dose, minimally toxic chemotherapeutic drugs has been explored recently for inoperable cancers where treatment options are limited or logistics are limited. This has been termed metronomic chemotherapy, as this resembles a metronome. Metronomic chemotherapy works by inhibiting tumor vasculature, thereby reducing tumor resistance to chemotherapeutic drugs [7]. Recently published research suggests that metronomic chemotherapy works through multiple mechanisms, including immunomodulation via the inhibition of T-regulatory cells, induction of tumor dormancy, and senescence, among others [8].

In developing countries like India, this approach has helped people in remote parts of the country with limited resources to access cancer care despite frequent visits to far-off hospitals or cancer care centers. Metronomic chemotherapy is relatively less expensive and avoids frequent hospital visits, as it carries a better toxicity profile due to the lower dose used per administration.

The ideal regimen, dose, and schedule of metronomic chemotherapy have been explored in various studies. Most studies on metronomic chemotherapy in head and neck cancers have chosen methotrexate and celecoxib as the regimen, with dose schedules varying between these studies. Patil et al. explored the optimum biological dose of methotrexate in locally advanced head and neck cancers to be 9 mg/m² in a phase 1 study [9]. The most commonly used regimens, methotrexate and celecoxib, were also the backbone of our research. No head-to-head comparison between dual and triple metronomic chemotherapy has been reported in the literature.

Hence, in this study, we aimed to compare the results between dual (methotrexate and celecoxib) and triple (methotrexate, celecoxib, and erlotinib) metronomic chemotherapy in patients with locally advanced, recurrent, and metastatic head and neck cancers at a tertiary care hospital in the northwestern part of India.

## Materials and methods

Study design

This retrospective study analyzed the clinical, demographic, and treatment-related characteristics of patients with advanced head and neck squamous cell carcinoma (HNSCC) treated with oral metronomic chemotherapy (OMT) using oral methotrexate 40 mg/m² once weekly for three weeks a month, celecoxib 200 mg twice daily, and erlotinib 150 mg once daily at our hospital between 2019 and 2024. Patients received no more than one prior line of systemic therapy before OMT. Palliative radiotherapy, delivered at a standard dose of 30 Gy in 10 fractions, was administered to patients who fulfilled the criteria for palliative radiotherapy.

Setting

The study was conducted at the Department of Radiation Oncology, All India Institute of Medical Sciences, Jodhpur. Data were collected from electronic medical records and patient documents in the outpatient department setting. The study was approved by the Institutional Ethics Committee, All India Institute of Medical Sciences, Jodhpur (approval number: AIIMS/IEC/2019-20/991).

Participants

Inclusion Criteria

We included patients aged more than 18 years with pathologically proven HNSCC, who were planned for palliative treatment, had an Eastern Cooperative Oncology Group Performance Status of 0-2, and were not planned for intravenous chemotherapy.

Exclusion Criteria

We excluded patients who are eligible for radical treatment, those with hematological malignancies such as non-Hodgkin’s lymphoma, those unwilling to undergo close follow-up, patients with any medical condition prohibiting the use of methotrexate or celecoxib (severely deranged liver function tests/myelosuppression, etc.), and those deemed unfit for radical treatment but unwilling to undergo metronomic chemotherapy.

Selection Criteria for Palliative Radiotherapy

We included patients with biopsy-proven HNSCC with the following clinically significant symptoms: (1) ulcerative or fungating mass affecting quality of life; (2) uncontrolled pain on oral pain medications (non-steroidal anti-inflammatory drugs + opioids + adjuvants); (3) bleeding from the tumor mass; (4) foul-smelling discharge not resolved with antibiotics and wound dressing; (5) large tumor mass causing significant mass effect on surrounding tissues; and (6) dysphagia Grade 3 or more due to tumor mass.

Patients diagnosed with locally advanced or metastatic HNSCC and who received at least one month of dual or triple oral metronomic chemotherapy between January 2019 and December 2024 were included.

Data collection

Data on patient demographics, clinical characteristics, and treatment details were extracted from medical records. The primary endpoints were OS and PFS. Secondary endpoints included the assessment of toxicities and other factors affecting survival.

Statistical analysis

Descriptive statistics were used to summarize patient characteristics. Comparative analyses were performed between the dual and triple chemotherapy groups using chi-square tests and Fisher’s exact tests for categorical variables and the Wilcoxon-Mann-Whitney test for continuous variables. Survival analysis was conducted using Kaplan-Meier curves, restricted mean survival times, and log-rank tests, while hazard ratios (HRs) were generated using Cox proportional hazard regression with time-adjusted covariates. Multivariate analysis was performed using Cox proportional hazards regression on factors that were significant in the univariate survival analysis, and interaction testing was performed to attest to their independent significance. Missing data were minimal in this study and, whenever present, were replaced with the median value of the study group to avoid bias. Sensitivity analysis was done before the formulation of the results. Statistical analysis was performed using SPSS version 26 (IBM Corp., Armonk, NY, USA) and Microsoft Excel (Microsoft Corp., Redmond, WA, USA).

## Results

The baseline characteristics of patients receiving dual (n = 45) and triple OMT (n = 52) are summarized in Table 1. The age distribution was comparable between the groups, with 53.3% of patients in the dual OMT cohort and 46.2% in the triple OMT cohort being younger than 50 years (p = 0.54). The majority of patients were female in both groups (77.8% vs. 76.9%; p = 1). The most common primary site was the oral cavity (62.2% in dual OMT vs. 69.2% in triple OMT), followed by the oropharynx, with no statistically significant difference across subsites (p = 0.5). Indications for therapy included locally advanced disease (64.4% vs. 63.5%) and recurrent/residual disease (35.6% vs. 26.9%), again without a significant difference (p = 0.66). The presence of metastasis was also balanced (20% vs. 21.2%; p = 1). However, prior receipt of chemotherapy was more common in the dual OMT group (64.4% vs. 40.4%; p = 0.026). Prior receipt of radiotherapy as either definitive or adjuvant was higher in the dual OMT group (31.3%) compared to the triple OMT group (23.1%) (p = 0.48). However, palliative radiation was significantly more frequent in the triple OMT group (88.5% vs. 62.2%; p = 0.001). Importantly, the median follow-up was markedly longer in the dual OMT cohort (20.2 months) compared with the triple OMT cohort (7.6 months), with high statistical significance (p < 0.00001).

**Table 1:** Baseline characteristics of patients in the dual and triple OMT groups. *: All except one patient (8 Gy in one fraction) received 30 Gy in 10 fractions as the palliative radiotherapy dose. OMT: oral metronomic therapy

Parameters	Dual OMT (n = 45)	Triple OMT (n = 52)	P-value
Age			
<50 years	24 (53.3%)	24 (46.2%)	0.54
>50 years	21 (46.7%)	28 (53.8%)	
Gender			
Male	10 (22.2%)	12 (23.1%)	1.00
Female	35 (77.8%)	40 (76.9%)	
Subsites			
Oral cavity	28 (62.2%)	36 (69.2%)	0.5
Oropharynx	9 (20.0%)	7 (13.5%)	
Nasopharynx	0 (0%)	1 (1.9%)	
Larynx	4 (8.9%)	1 (1.9%)	
Hypopharynx	1 (2.2%)	3 (5.8%)	
Unknown primary	3 (6.7%)	3 (5.8%)	
Other head and neck cancers	0 (0%)	1 (1.9%)	
Indication			
Locally advanced	29 (64.4%)	33 (63.5%)	0.66
Recurrent/Residual disease	16 (35.6%)	14 (26.9%)	
Presence of metastasis			
No	36 (80.0%)	40 (76.9%)	1.00
Yes	9 (20.0%)	11 (21.2%)	
Receipt of prior chemotherapy			
Yes	29 (64.4%)	21 (40.4%)	0.026
No	16 (35.6%)	30 (57.7%)	
Receipt of prior radiotherapy (definitive or adjuvant)			
Yes	14 (31.3%)	12 (23.1%)	0.48
No	31 (68.7%)	40 (76.9%)	
Receipt of palliative radiation*			
Yes	28 (62.2%)	46 (88.5%)	<0.0001
No	17 (37.8%)	6 (11.5%)	
Median follow-up	20.2 (17.6–22.8) months	7.6 (6.1–9.2) months	<0.00001

Overall survival analysis

**Figure 1:**
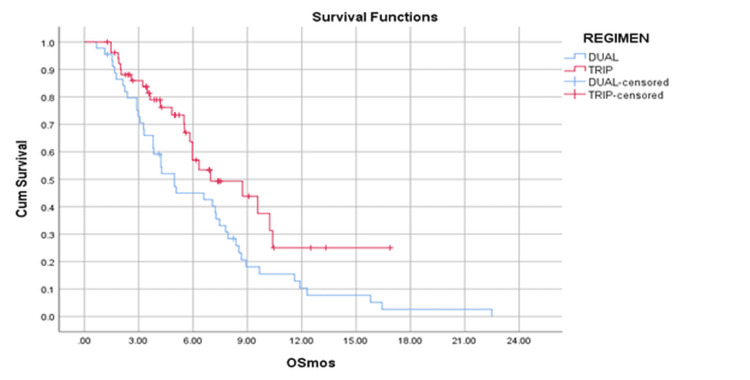
Kaplan-Meier survival curves showing OS for patients receiving dual OMT versus triple OMT. The blue line represents the dual OMT group, and the red line represents the triple OMT group. OS is defined as the time from initiation of OMT to death from any cause. OMT: oral metronomic therapy; OS: overall survival

The median follow-up was 20.2 months in the dual OMT cohort and 7.6 months in the triple cohort arm (p < 0.0001). There were 42 and 23 deaths each in the dual and triple OMT cohorts. The median OS was 4.9 months (95% confidence interval (CI) = 3.7-6.3) for patients receiving dual OMT and 6.9 months (95% CI = 3.3-10.6) for those receiving triple OMT (p =0.023). The six-month and one-year OS rates for the dual OMT cohort were 45% and 11%, respectively, while those for the triple OMT cohort were 56% and 26%, respectively (Figure 1). The only factors that improved OS on univariate analysis were the choice of triple OMT over dual OMT (HR = 0.56, 95% CI = 0.33-0.93, p = 0.025) and receipt of palliative radiotherapy (HR = 0.42, 95% CI = 0.25-0.72, p = 0.001) (Table 2). On multivariate analysis, receiving palliative radiotherapy was the only factor that favorably impacted OS (HR = 0.49, 95% CI = 0.28-0.87, p = 0.015).

**Table 2:** Factors affecting overall survival. CI: confidence interval; OMT: oral metronomic chemotherapy; RT: radiotherapy

Variable	Hazard ratio	95% CI of hazard ratio	P-value
Age	0.99	0.97-1.02	0.899
Gender (reference: female)	0.83	0.47-1.47	0.539
Triple OMT (reference: dual OMT)	0.56	0.33-0.93	0.025
Oral (reference: other subsites)	0.57	0.12-2.69	0.48
Indication for use: locally advanced disease (reference: recurrent/residual)	0.68	0.39-1.16	0.15
Receipt of prior systemic chemotherapy before OMT	0.70	0.43-1.15	0.16
Receipt of palliative RT before or after OMT	0.42	0.25-0.72	0.001
Sequence of palliative RT before OMT (reference: after OMT)	0.83	0.40-1.70	0.61

Progression-free survival analysis

**Figure 2:**
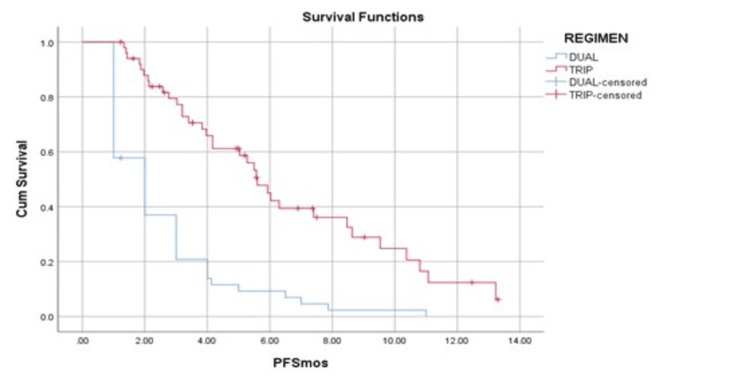
Kaplan-Meier survival curves showing PFS of patients receiving dual OMT versus triple OMT. The blue line represents the dual OMT group, and the red line represents the triple OMT group. PFS is defined as the time from initiation of OMT to documented disease progression or death from any cause. OMT: oral metronomic therapy; PFS: progression-free survival

The median PFS was two months (95% CI = 1.3-2.7) for patients receiving dual OMT and 5.6 months (95% CI = 4.8-6.4) for those receiving triple OMT (p < 0.0001). The six-month and one-year PFS rates for the dual OMT cohort were 12% and 2.5%, respectively, while they were 60% and 12.5% for the triple OMT cohort (Figure 2). Patients who received triple OMT (HR = 0.29, 95% CI = 0.18-0.47, p < 0.0001) or palliative radiotherapy (HR = 0.39, 95% CI = 0.24-0.67, p = 0.00049) had better median PFS compared to the others (Table 3). Multivariate analysis revealed that the triple OMT regimen (HR = 0.31, 95% CI = 0.19-0.51, p = 0.000004) and receipt of palliative radiotherapy (HR = 0.51, 95% CI = 0.30-0.87, p = 0.013) both significantly improved the median PFS.

**Table 3:** Factors affecting progression-free survival. CI: confidence interval; OMT: oral metronomic chemotherapy; RT: radiotherapy

Variable	Hazard ratio	95% CI of hazard ratio	P-value
Age	0.99	0.98-1.01	0.521
Gender (reference: female)	0.99	0.58-1.71	0.98
Triple OMT (reference: dual OMT)	0.29	0.18-0.47	<0.00001
Oral (reference: other subsites)	0.60	0.24-1.52	0.29
Indication for use: locally advanced disease (reference: recurrent/residual)	0.68	0.39-1.16	0.15
Receipt of prior systemic chemotherapy before OMT	0.83	0.53-1.31	0.43
Receipt of palliative RT before or after OMT	0.39	0.24-0.67	0.00049
Sequence of palliative RT before OMT (reference: after OMT)	1.32	0.71-2.45	0.38

To evaluate whether the benefit of triple OMT differed according to palliative radiotherapy status, an interaction term (regimen × radiotherapy) was introduced. The interaction was not statistically significant (p = 0.35), indicating that the advantage of triple OMT over dual OMT was consistent regardless of palliative radiotherapy. The stratum-specific HRs for dual versus triple OMT were 2.9 among patients who received palliative radiotherapy and 1.6 among those who did not, confirming a similar direction of benefit across radiotherapy.

Acute toxicity (Common Terminology Criteria for Adverse Events, version 5.0)

Oral mucositis was the most frequent adverse event, occurring in 46.2% of patients in the triple OMT group compared to 31.1% in the dual OMT group, with Grade ≥3 observed in 5.7% and 4.4% of patients, respectively (Table 4). Rash was reported in 5.8% of patients in the triple OMT group (Grade ≥3 in 1.9%), whereas only one (2.2%) patient in the dual OMT group had a mild rash. Diarrhea occurred in 15.4% of patients in the triple OMT group and 11.1% in the dual OMT group, with one patient in the triple OMT group reporting Grade ≥3 severity. Hand-foot syndrome was noted only in the triple OMT group, affecting 3.8% of patients, though no severe cases were reported. Overall, both regimens were tolerated well, with Grade ≥3 toxicities being relatively infrequent.

**Table 4:** Comparison of acute toxicity between triple versus dual metronomic chemotherapy. OMT: oral metronomic chemotherapy

Clinical toxicity	Triple OMT (n = 52)		Dual OMT (n = 45)	
	Any grade, n (%)	Grade ≥3, n (%)	Any grade, n (%)	Grade ≥3, n (%)
Oral mucositis	24 (46.2%)	3 (5.7%)	14 (31.1%)	2 (4.4%)
Rash	3 (5.8%)	1 (1.9%)	1 (2.2%)	0
Diarrhea	8 (15.4%)	1 (1.9%)	5 (11.1%)	0
Hand-foot syndrome	2 (3.8%)	0	0	0

## Discussion

Our study provides novel insights into the comparative efficacy of dual versus triple OMT in patients with advanced HNSCC. The results indicate that the addition of erlotinib to the standard dual regimen of methotrexate and celecoxib significantly improves OS and PFS. To our knowledge, there are no existing studies directly comparing dual versus triple OMT in HNSCC. This highlights the uniqueness of our research in evaluating the incremental benefit of adding erlotinib to the established dual OMT regimen. While multiple studies have assessed the efficacy of individual OMT agents or dual combinations [10], our study is the first to compare the clinical outcomes of dual versus triple OMT directly.

In resource-limited settings, such as India, the cost-effectiveness of treatment modalities is a critical factor. A study by Patil et al. [9] demonstrated that dual OMT is a cost-effective alternative to intravenous cisplatin, with a median OS of 7.5 months compared to 6.1 months for cisplatin.

Our findings align with previous studies that have explored the efficacy of triple OMT regimens. A phase I/II study on palliative triple metronomic chemotherapy in platinum-refractory or early-failure oral cancer from Tata Medical Hospital, India, reported a median OS of 6.7 months [11], which is similar to the median OS observed in our study (6.9 months). The trend of improved survival with triple OMT compared to conventional chemotherapy is consistent across studies, highlighting its potential role in managing advanced HNSCC.

A recent phase III randomized study [12] further reinforces the clinical utility of triple OMT by demonstrating its efficacy when combined with standard paclitaxel-carboplatin chemotherapy. Conducted in an Indian cohort of 238 patients with advanced HNSCC, the addition of triple OMT significantly improved both median OS (8.3 vs. 6.1 months; p = 0.00011) and PFS (7.6 vs. 3.5 months; p < 0.0001) compared to chemotherapy alone. Importantly, this regimen maintained good tolerability and quality of life outcomes, with manageable toxicity profiles. These findings support the broader application of triple OMT in resource-limited settings, where access to biologics or immunotherapy remains a challenge.

Additionally, the study by Patil et al. [13] on low-dose immunotherapy plus triple metronomic chemotherapy for head and neck cancer demonstrated that when combining low-dose nivolumab with triple metronomic chemotherapy, there was a statistically significant survival benefit (6.7 months in the triple metronomic arm vs. 10.1 months in the triple metronomic + nivolumab arm, p = 0.0052) suggesting the synergistic potential of combining immune checkpoint inhibitors with OMT [14].

In our study comparing toxicity profiles of dual versus triple OMT in locally advanced HNSCC, the incidence of treatment-related adverse events was low in both arms, with a slightly higher frequency of toxicities observed in the triple OMT cohort. Oral mucositis was the most common toxicity, reported in 46.2% of patients in the triple arm versus 31.1% in the dual arm, although Grade 3 or higher mucositis remained infrequent (5.7% vs. 4.4%). Other toxicities, including rash, diarrhea, and hand-foot syndrome, were mild and uncommon in both groups. Notably, hand-foot syndrome occurred only in the triple OMT group, albeit at a low rate (3.8%). The overall safety profile of both regimens appears favorable, with no significant increase in high-grade toxicity in the triple arm, suggesting that the addition of a third agent may be feasible from a tolerability standpoint. However, a modest increase in low-grade mucositis and gastrointestinal side effects should be anticipated. This finding aligns with the existing literature and does not reveal any significant deviation from other studies, despite using a comparatively higher dose of methotrexate in our regimen. However, the higher dose of methotrexate does not translate into a better OS or PFS, nor does it increase the incidence of Grade 3 or higher toxicities. This requires further exploration in randomized settings, and additional toxicity parameters should be assessed.

In India, the approximate monthly cost of OMT (methotrexate, celecoxib, and erlotinib) ranges from INR 5,000 to 7,000, whereas single-agent intravenous chemotherapy and immunotherapy come at a higher price. The per-cycle cost of nivolumab is five to six times higher than that of OMT, with additional out-of-pocket expenditures including hospitalization and travel expenses. The affordability and oral administration of OMT make it a viable option for patients who may not have access to more expensive therapies.

Both triple OMT and palliative radiotherapy independently conferred superior PFS, suggesting additive rather than synergistic effects. The absence of a statistically significant interaction (p = 0.35) implies that the efficacy of the triple regimen was maintained across patients who did and did not receive palliative radiotherapy. These findings support the use of triple OMT as an effective systemic option in the palliative setting, independent of prior or concurrent palliative radiation.

The role of palliative radiotherapy has been established in locally advanced, recurrent, and metastatic head and neck cancers. Palliative radiotherapy is often used for symptom palliation and improves quality of life [15]. The importance of palliative radiotherapy in improving OS in patients with advanced HNSCC has been well-documented [16]. A Surveillance, Epidemiology, and End Results database analysis [14] demonstrated that radiation significantly improves OS in metastatic HNSCC patients. This aligns with our findings, where receipt of palliative radiotherapy was associated with a significant improvement in both OS and PFS. The integration of palliative radiotherapy into treatment strategies could provide meaningful survival benefits for patients receiving OMT.

The integration of erlotinib into the dual OMT regimen offers a promising therapeutic strategy, particularly in settings where access to advanced therapies is limited. The oral administration and favorable cost profile further enhance its applicability in low-resource environments. Notably, the significant impact of palliative radiotherapy on OS and PFS suggests that a multimodal approach combining radiation and OMT may be the most effective strategy for patients with advanced HNSCC.

## Conclusions

This study comparing triple versus dual OMT in locally advanced, recurrent, and metastatic head and neck cancers showed that the addition of erlotinib significantly improves PFS, with minimal toxicity. Despite a higher dose of methotrexate used in this study, there was no significant OS or PFS benefit compared to other studies. There were no significantly higher Grade 3 or 4 toxicities with the increased dose of methotrexate. Receipt of palliative radiotherapy was also independently associated with an increase in OS and PFS.

## Appendices

**Table 5:** Supplementary data of the survival analysis of dual and triple OMT. OMT: oral metronomic therapy; OS: overall survival

Survival table for OS							
Regimen		Time	Status	Cumulative proportion surviving at the time		Number of cumulative events	Number of remaining cases
				Estimate	Standard error		
Dual	1	0.670	1.00	0.977	0.023	1	42
	2	1.130	1.00	0.953	0.032	2	41
	3	1.270	0.00	.	.	2	40
	4	1.530	1.00	0.930	0.039	3	39
	5	1.570	1.00	0.906	0.045	4	38
	6	1.670	1.00	0.882	0.050	5	37
	7	1.770	1.00	0.858	0.054	6	36
	8	2.130	1.00	0.834	0.057	7	35
	9	2.230	1.00	0.810	0.060	8	34
	10	2.370	1.00	0.787	0.063	9	33
	11	2.900	1.00	.	.	10	32
	12	2.900	1.00	0.739	0.068	11	31
	13	2.970	1.00	0.715	0.070	12	30
	14	3.070	1.00	0.691	0.071	13	29
	15	3.270	1.00	0.667	0.073	14	28
	16	3.300	1.00	0.644	0.074	15	27
	17	3.800	1.00	.	.	16	26
	18	3.800	1.00	0.596	0.076	17	25
	19	3.830	1.00	0.572	0.076	18	24
	20	4.130	0.00	.	.	18	23
	21	4.230	1.00	.	.	19	22
	22	4.230	1.00	0.522	0.077	20	21
	23	4.270	1.00	0.497	0.078	21	20
	24	4.970	1.00	.	.	22	19
	25	4.970	1.00	0.448	0.077	23	18
	26	6.600	1.00	0.423	0.077	24	17
	27	7.070	1.00	0.398	0.076	25	16
	28	7.230	1.00	0.373	0.076	26	15
	29	7.270	1.00	0.348	0.074	27	14
	30	7.470	1.00	0.323	0.073	28	13
	31	7.800	1.00	0.298	0.072	29	12
	32	7.930	1.00	0.274	0.070	30	11
	33	8.230	0.00	.	.	30	10
	34	8.370	1.00	0.246	0.068	31	9
	35	8.530	1.00	0.219	0.066	32	8
	36	8.670	1.00	0.192	0.063	33	7
	37	8.930	1.00	0.164	0.060	34	6
	38	9.670	1.00	0.137	0.056	35	5
	39	11.600	1.00	0.109	0.051	36	4
	40	11.900	1.00	0.082	0.045	37	3
	41	15.800	1.00	0.055	0.037	38	2
	42	16.430	1.00	0.027	0.027	39	1
	43	22.500	1.00	0.000	0.000	40	0
dual	1	1.900	1.00	0.833	0.152	1	5
	2	2.600	1.00	0.667	0.192	2	4
	3	4.830	1.00	0.500	0.204	3	3
	4	5.070	1.00	0.333	0.192	4	2
	5	9.570	1.00	0.167	0.152	5	1
	6	12.300	1.00	0.000	0.000	6	0
Triple	1	1.270	0.00	.	.	0	47
	2	1.470	1.00	.	.	1	46
	3	1.470	1.00	0.957	0.029	2	45
	4	1.670	0.00	.	.	2	44
	5	1.870	1.00	0.936	0.036	3	43
	6	2.000	1.00	0.914	0.041	4	42
	7	2.030	1.00	0.892	0.046	5	41
	8	2.270	0.00	.	.	5	40
	9	2.400	0.00	.	.	5	39
	10	2.500	0.00	.	.	5	38
	11	2.670	0.00	.	.	5	37
	12	3.230	1.00	0.868	0.050	6	36
	13	3.400	0.00	.	.	6	35
	14	3.430	0.00	.	.	6	34
	15	3.470	1.00	0.843	0.055	7	33
	16	3.570	0.00	.	.	7	32
	17	3.570	0.00	.	.	7	31
	18	3.630	1.00	0.815	0.060	8	30
	19	3.870	0.00	.	.	8	29
	20	4.000	0.00	.	.	8	28
	21	4.170	0.00	.	.	8	27
	22	4.200	1.00	0.785	0.065	9	26
	23	4.270	0.00	.	.	9	25
	24	4.970	0.00	.	.	9	24
	25	5.030	0.00	.	.	9	23
	26	5.230	0.00	.	.	9	22
	27	5.500	1.00	0.749	0.071	10	21
	28	5.530	1.00	0.714	0.076	11	20
	29	5.600	.00	.	.	11	19
	30	5.830	1.00	0.676	0.081	12	18
	31	5.970	1.00	.	.	13	17
	32	5.970	1.00	0.601	0.087	14	16
	33	6.170	0.00	.	.	14	15
	34	6.330	1.00	0.561	0.090	15	14
	35	6.900	0.00	.	.	15	13
	36	6.900	0.00	.	.	15	12
	37	6.970	1.00	0.514	0.094	16	11
	38	7.370	0.00	.	.	16	10
	39	7.430	0.00	.	.	16	9
	40	7.530	0.00	.	.	16	8
	41	8.730	1.00	0.450	0.102	17	7
	42	9.070	0.00	.	.	17	6
	43	10.230	1.00	0.375	0.109	18	5
	44	10.400	1.00	0.300	0.110	19	4
	45	10.470	0.00	.	.	19	3
	46	12.500	0.00	.	.	19	2
	47	13.330	0.00	.	.	19	1
	48	16.870	0.00	.	.	19	0
